# miR-30b-3p Inhibits Proliferation and Invasion of Hepatocellular Carcinoma Cells *via* Suppressing PI3K/Akt Pathway

**DOI:** 10.3389/fgene.2019.01274

**Published:** 2019-12-13

**Authors:** Dongkai Gao, Zumo Zhou, Heqing Huang

**Affiliations:** Department of Infectious Diseases, Zhuji People’s Hospital of Zhejiang Province, Shaoxing, China

**Keywords:** miR-30b-3p, miRNA, hepatocellular carcinoma, TRIM27, PI3K/Akt

## Abstract

**Background:** The micro-RNA miR-30b-3p has been reported to play a crucial role in several cancers. However, the biological function of miR-30b-3p in hepatocellular carcinoma (HCC) is still unknown.

**Methods:** RT-qPCR was employed to determine the expression of miR-30b-3p in HCC tissues and cells. The MTT assay, colony formation assay, and cell migration and invasion assay were employed to evaluate the role of miR-30b-3p in HCC cells. A dual-luciferase reporter assay was employed to verify the target of miR-30b-3p. Western blotting was employed to determine the expression of key molecular signal transducers along TRIM27-PI3K/Akt axis.

**Results:** Expression of miR-30b-3p was markedly decreased in HCC tissues and cells and positively correlated with higher overall survival. Moreover, miR-30b-3p overexpression significantly repressed cell viability, proliferation, migration, and invasion of HCC cells *in vitro*. Notably, we demonstrated that miR-30b-3p directly bound to the 3′-untranslated region of tripartite motif containing 27 (TRIM27) mRNA by downregulating the expression of TRIM27, which was demonstrated to be negatively correlated with miR-30b-3p expression. TRIM27 was demonstrated to have an oncogenic role in HCC cells by enhancing cell viability, proliferation, migration, and invasion. Finally, the miR-30-3p-TRIM27-PI3K/Akt axis was shown to play a crucial role in HCC cells *in vitro*.

**Conclusion:** Our results indicated that miR-30-3p might act as a new biomarker for the future diagnosis and treatment HCC.

## Introduction

Hepatocellular carcinoma (HCC) is still one of the deadliest and most prevalent human malignant cancers worldwide. According to the global cancer statistics in 2018, HCC is estimated to be the fourth major cause of cancer death and the sixth most commonly diagnosed cancer (Bray et al., 2018; Siegel et al., 2018; Ferlay et al., 2019). Statistical data indicate that approximately 782,000 deaths and 841,000 new cases are reported every year. Although much effort has been made to find a cure for HCC, such as surgical resection, chemotherapy, radiotherapy, and liver transplantation, the prognosis is still not satisfactory. Therefore, it is urgent for us to understand the underlying mechanisms of HCC carcinogenesis in depth and to further uncover novel targets for the diagnosis and treatment of HCC.

MicroRNAs (miRNAs) are a class of non-coding RNAs approximately 19-22 nucleotides in length. Recent studies reported that miRNAs regulate gene expression *via* binding to the 3′-untranslated region (UTR) of target mRNA at the post-transcriptional level (Bartel, 2004; Ponting et al., 2009). Increasing evidence has been uncovered that almost all diseases are related to the regulation of miRNAs, including cancers (Garzon et al., 2006; Bartels and Tsongalis, 2009; Garzon et al., 2009; Ryan et al., 2010; Chan and Tay, 2018). It has been demonstrated that miR-30b-3p was downregulated in primary prostate cancer (PCa) and metastatic castration resistant PCa and can directly inhibit androgen receptor and PCa cell proliferation (Kumar et al., 2016). Kung et al. reported that miR-30b-5p can inhibit epithelial-mesenchymal transition (EMT) and suppress cell migration and invasion in PCa through EGF/Src signalling (Kao et al., 2014). In addition, Zeng et al. demonstrated that miR-30b-3p was elevated in glioma cells, overexpression of miR-30b-3p could act in an oncogenic role *via* activation of the Akt pathway (Jian et al., 2019). However, the role of miR-30b-3p in HCC remains largely unclear.

In this study, we explored the expression pattern of miR-30b-3p in HCC tissues and cell lines and investigated the function of miR-30b-3p in HCC cells. Furthermore, bioinformatics analysis and dual-luciferase reporter assay were used to identify potential targets of miR-30b-3p. Moreover, we found that miR-30b-3p inhibited the proliferation and invasion of HCC cells by suppressing TRIM27 expression to inactivate the PI3K/Akt pathway.

## Materials and Methods

### Tissue Samples

The study included 50 paired HCC tissues and their matched non-tumour tissues that were collected form Zhuji People’s Hospital of Zhejiang Province between July 2014 and July 2019. The ethics committee of the Zhuji People’s Hospital of Zhejiang Province approved the study (No: 20180224). The tissue samples were snap-frozen in liquid nitrogen and stored at −70°C before use.

### Cell Culture and Transfection

Human HCC cell lines (Huh7 and HepG2) and a human normal liver cell line (LO2) were obtained from the American Type Culture Collection. Huh7, HepG2, and LO2 cells were cultured in Dulbecco’s Modified Eagle Medium (Thermo Fisher Scientific, USA) containing 10% fetal bovine serum (FBS, Thermo Fisher Scientific, USA) and cultured in an incubator maintained at 37°C with 5% CO_2_.

The miR-30b-3p mimics (5′-CUGGGAGGUGGAUGUUUAUUC-3′) or anti-miR-30b-3p (5′-GAAGUAAACAUCCACCUCCCAG-3′) and their negative control (miR-NC mimics, 5′-UUCUCCGAACGUGUCACGUTT-3′ and anti-miR-NC, 5′-ACGUGACACGUUCGGAGAATT-3′) or relative plasmids were transfected into HCC cells using Fugene HD (Roche) in OPTI-MEM media (Thermo Fisher Scientific, USA).

### RNA Extraction and Real-Time Quantitative Polymerase Chain Reaction (RT-qPCR)

Extraction of the total RNA of HCC tumour and normal tissue samples, as well as treated and non-treated HCC cells was performed by using TRIzol reagent (Wanlei Bio, China). Then, the concentration of extracted RNA was determined using a NanoPhotometer spectrophotometer (Implen, Germany). For cDNA synthesis, 2 μg total RNA was added as a template for reverse transcription using a TRUEscript One Step RT-PCR Kit (Aidlab Biotechnologies, China). An ABI7500 system was employed to quantify the levels of miR-30b-3p and TRIM27 in HCC tissues and cells by using PC60-2 x SYBR Green qPCR Mix (Low ROX) (Aidlab Biotechnologies, China). The primer sequences used were as follows: GAPDH, F: 5ʹ- CTGGGCTACACTGAGCACC -3ʹ, R: 5ʹ-AAGTGGTCGTTGAGGGCAATG-3ʹ; U6, F: 5ʹ- TGCGGGTGCTCGCTTCGGCAGC-3ʹ, R: 5ʹ- -CCAGTGCAGGGTCCGAGGT -3ʹ, RT: 5′-GTCGTATCCAGTGCAGGGTCCGAGGTATTCGCACTGGATACGACAAAATATGGAAC -3′; miR-30b-3p, F: 5ʹ- TGCGGAGAGGTTGCCCTTGGTGA −3ʹ, R: 5ʹ- TGCGGGTGCTCGCTTCGGCAGC -3ʹ, RT: 5ʹ- GTCGTATCCAGTGCAGGGTCCGAGGTGCACTGGATACGACGAATTCAC-3ʹ; TRIM27, F: 5ʹ- TGAGCCTAACCCAGATGGAGA-3ʹ, R: 5ʹ- GGCCAAGTCTAGCTCCTCAAG-3ʹ. TRIM27 mRNA level and miR-30b-3p expression levels were normalized using GAPDH and U6 as the internal control, respectively. The 2−ΔΔCt method was used to quantify the transcript level of TRIM27 and miR-30b-3p.

### MTT Assay

Huh7 and HepG2 cells were transfected with miR-30b-3p mimics or anti-miR-30b-3p and their respective negative controls (miR-NC mimics and anti-miR-NC) or relative plasmids for 24 h. Then, cells were seeded in 96-well plates to obtain a cell density of 3 × 103 per well. Each group contained five duplicate wells. MTT (Sigma, USA) was then added to the 96-well plates to measure cell viability at 0, 24, 48 and 72 h, respectively. The absorbance (OD570) value was measured using a Microplate reader (Bio-Rad, USA).

### Colony Formation Assay

HCC cells (3 × 10^2^) that were transfected with relative miR-30b-3p mimics or anti-miR-30b-3p and their negative controls or plasmids were seeded into 6-well plates and incubated at 37°C for 2 weeks. Then, 4% formaldehyde was used to fix for 30 min and 0.25% crystal violet was used to stain the colonies. Colonies of more than 50 cells were counted under a microscope.

### Cell Migration and Invasion Assay

The Transwell cell migration and invasion assay was employed to assess cell migratory and invasive abilities. Cells were seed into 24-well Transwell Boyden chambers (8.0 μm pore size; Costar, Cambridge, MA) based on the manufacturer’s instructions. Briefly, for the Transwell cell migration assay, 5 × 10^4^ cells were suspended in 200 µl DMEM without FBS and added to the upper chambers, then 600 μl DMEM containing 20% FBS was added into the lower chambers and incubated for 2 h at 37°C. For the Transwell cell invasion assay, 8 × 10^4^ cells were suspended in 200 µl DMEM without FBS and added to the upper chambers, which had been filled with 40 µl Matrigel. Thereafter, non-invasive cells on the inner surfaces of the upper chambers were gently scraped off using a cotton swab. Invasive cells were fixed with 100% methanol, stained with 0.5% crystal violet solution, and washed with 1× PBS. The cell number was counted under a light microscope (Nikon, Japan).

### Dual-Luciferase Reporter Assay

Cells (3 × 10^4^) were transferred to 24-well plates and cultured for 24 h. The cells were then co-transfected with pRL-TK plasmid, pmir-luc-TRIM27-3′-UTR, or pmir-luc-TRIM27-3′-UTR mut plus miR-30b-3p mimics or inhibitors, and their corresponding negative controls. The luciferase reporter assay kit (Promega) was used to measure luciferase activity.

### Western Blot

RIPA lysis buffer (Wanlei Bio, China) containing protease inhibitor cocktail (Roche) was used to extract the protein from the harvested cells. Then, the protein samples were loaded onto 10% SDS-PAGE gels and transferred onto PVDF membranes (Millipore, USA). After blocking with 5% BSA, the PVDF membranes were incubated with primary antibodies overnight at 4°C and probed with horseradish peroxidase (HRP)-conjugated secondary antibodies for 2 h at room temperature. Thereafter, enhanced chemiluminescence (ECL) (Wanlei Bio, China) was used to detect the protein bands. The following antibodies were included: GAPDH (WL01114, 1:2000 dilution, Wanlei Bio, China); TRIM27 (ab154931, 1:500 dilution, Abcam, USA); Akt (ab179463, 1:1000 dilution, Abcam, USA); p-Akt (ab192623, 1:1000 dilution, Abcam, USA); Goat Anti-mouse IgG H&L (HRP) (ab205719, 1:2000 dilution, Abcam, USA) and Goat Anti-Rabbit IgG H&L (HRP) secondary antibody (ab6702, 1:2000 dilution, Abcam, USA).

### Immunohistochemical Analysis

Immunohistochemistry (IHC) was performed according to a previously reported method (Liu et al., 2017). Briefly, 4% formalin was used to fix the tissues for 2 days. Then the tissues were embedded using paraffin and cut into 5-micron slices. The slices underwent antigen retrieval after peeling and rehydration and were blocked with 3% H_2_O_2_. Subsequently, the slides were incubated overnight at 4°C with antibodies against p-Akt (1:100 dilution, Abcam, USA). Slides were then incubated with Goat Anti-mouse IgG H&L secondary antibody coupled with biotin at room temperature for 15 min. Subsequently, the streptavidin-biotin complex and DAB were added to the slides. Finally, hematoxylin was used to counterstain the slides.

### Statistical Analysis

All experiments were repeated at least three times for statistical analyses. Data are presented as the mean ± SD. Student’s *t* test and ANOVA (with a Bonferroni *post hoc* test) were used to compare the mean values between the two groups and the mean values greater than or equal to three groups. Differences were considered statistically significant at P < 0.05.

## Results

### miR-30b-3p Was Dysregulated in HCC

**Figure 1:**
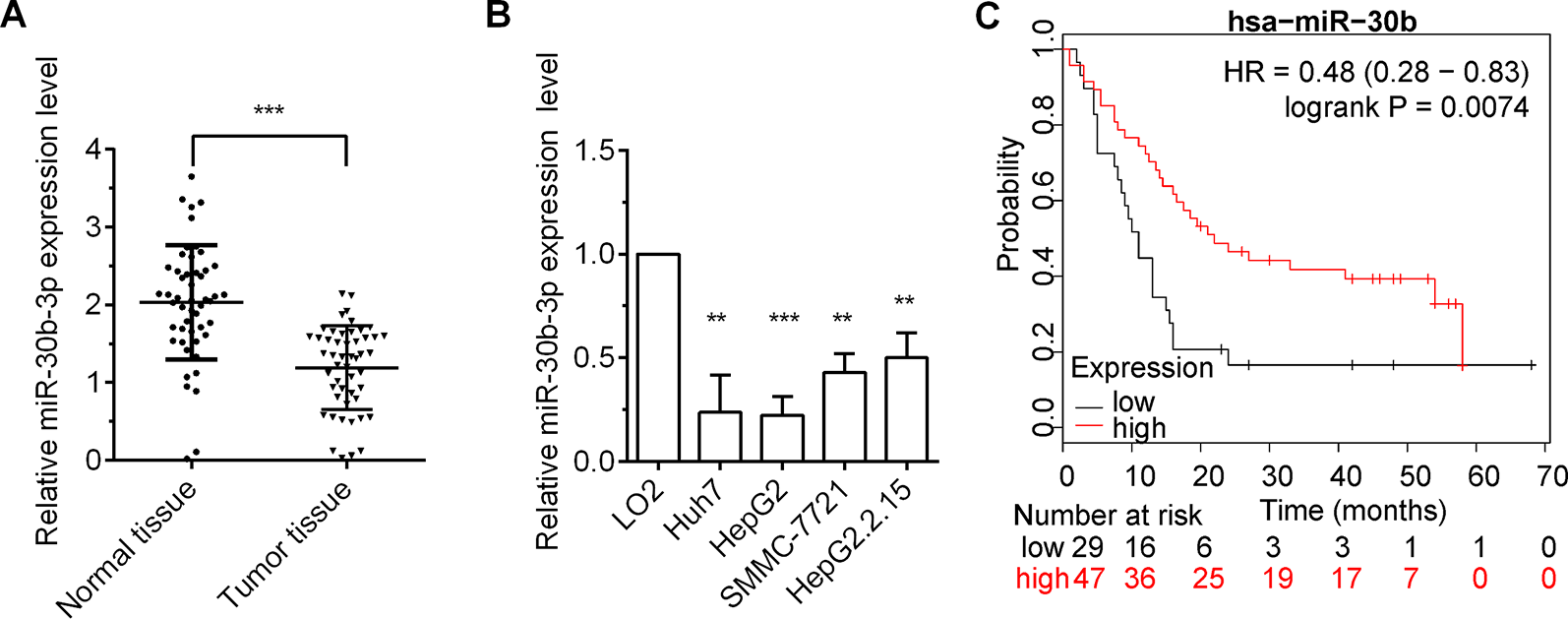
miR-30b-3p expression was low in HCC tissues and cells. **(A)** RT-qPCR detected the relative expression level of miR-30b-3p in 50 paired HCC tissues and their matched-non-carcinoma tissues. **(B)** RT-qPCR detected the relative expression level of miR-30b-3p in HCC cell lines and normal live cell line. **(C)** The Kaplan–Meier plotter online tool was used to assess the effect of miR-30b-3p on survival in HCC (http://kmplot.com/analysis/). Data were expressed as mean ± SD, ^**^p < 0.01; ^***^p < 0.001.

To investigate the role miR-30b-3p in HCC, we first measured the expression of miR-30b-3p in HCC tumor tissues and cell lines by RT-qPCR. The results showed that miR-30b-3p was downregulated markedly in HCC tumour tissues compared with matched non-carcinoma tissues (**Figure 1A**). In addition, the relative expression of miR-30b-3p in four HCC cell lines, Huh7, HepG2, SMMC-7721 and HepG2.2.15, was markedly reduced compared with that in the normal liver cell line LO2 (**Figure 1B**). Moreover, the effect of miR-30b-3p on survival in HCC was assessed using the Kaplan–Meier plotter online tool (http://kmplot.com/analysis/). As shown in **Figure 1C**, HCC patients with high expression of miR-30b-3p exhibited longer survival than those with low expression of miR-30b-3p.

### miR-30b-3p Repressed HCC Cell Proliferation, Migration, and Invasion

**Figure 2:**
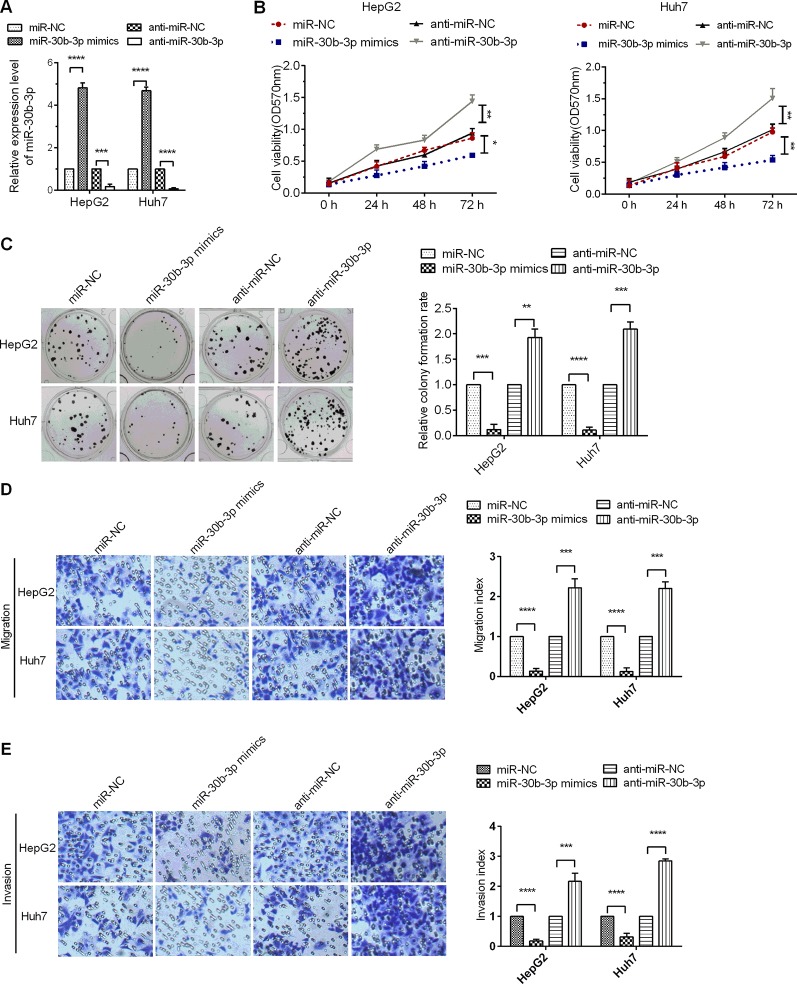
miR-30b-3p overexpression inhibited HCC cell proliferation and metastasis. **(A)** RT-qPCR was used to detect the expression levels of miR-30b-3p after transfection with miR-30b-3p mimics or its anti-miR-30b-3p. **(B)** MTT assay was used to detect the cell viability of HCC cells transfected with miR-30b-3p mimics or anti-miR-30b-3p. **(C)** A colony formation assay was performed on HCC cells after transfection as indicated. **(D**, **E)** Representative photographs of cell migration and invasion of HCC cells after transfection as indicated. Data were expressed as mean ± SD, ^*^p < 0.05; ^**^p < 0.01; ^***^p < 0.001; ^****^p < 0.0001.

To assure the role miR-30b-3p in HCC cells, miR-30b-3p mimics or anti-miR-30b-3p was transfected into Huh7 and HepG2 cells. The expression of miR-30b-3p was first detected in HCC cells to verify the efficacy of miR-30b-3p mimics or anti-miR-30b-3p by RT-qPCR assays (**Figure 2A**). Next, MTT assay results revealed that miR-30b-3p overexpression reduced cell viability in HCC cells, whereas downregulation of the expression of miR-30b-3p enhanced cell viability (**Figure 2B**). Further, the proliferative effect of HCC cells transfected with miR-30b-3p mimics was markedly inhibited when compared with those transfected with miRNA NC mimics, whereas the proliferative effect of HCC cells was significantly enhanced when transfected with anti-miR-30b-3p (**Figure 2C**). Then, to investigate the role of miR-30b-3p in HCC cell migration and invasion, the Transwell migration and invasion assay revealed that, compared with the miRNA NC mimics group, migration and invasion in the miR-30b-3p mimics group were decreased remarkably, whereas the migratory and invasive activities of the anti-miR-30b-3p group showed a significant increase (**Figures 2D**, **E**). These results implied that miR-30b-3p might play the role of a tumour suppressor gene in HCC cells.

### miR-30b-3p Repressed the mRNA Level of TRIM27

**Figure 3:**
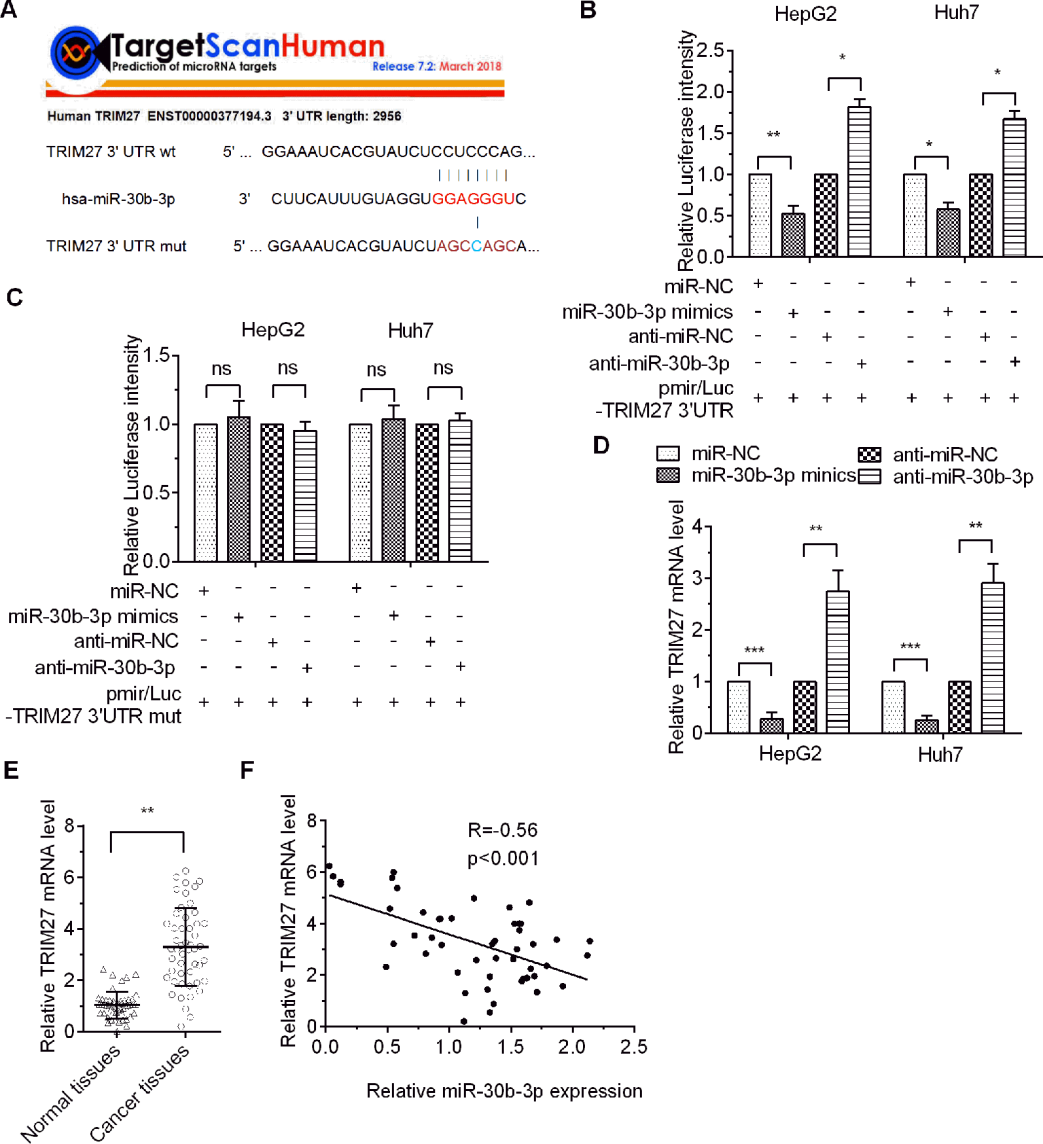
TRIM27 was the direct target of miR-30b-3p. **(A)** The indicated sequences of TRIM27 targeted by miR-30b-3p were predicted using TargetScan 7.1, and the wildtype 3′-UTR or the mutational 3′-UTR of TRIM27 mRNA are shown. **(B**, **C)** Dual-luciferase report assay was used to assess relative luciferase activity for wildtype TRAF3 3′-UTR or mutant 3′-UTR. **(D)** RT-qPCR detected the relative mRNA levels of TRIM27 in different transfected groups. **(E)** RT-qPCR detected the mRNA levels of TRIM27 in 50 paired HCC tissues and their matched non-carcinoma tissues. **(F)** miR-30b-3p expression and TRIM27 mRNA levels showed a negative correlation. All the experiments were repeated three times. ^*^p < 0.05; ^**^p < 0.01; ^***^p < 0.001; ns, not significant.

To screen for putative targets of miR-30b-3p, the results obtained with the online bioinformatics software TargetScan Human 7.2 revealed that the 3′-UTR of TRIM27 (also called RFP) mRNA has specific binding sites for miR-30b-3p seed sequences, indicating that TRIM27 might be a potential target of miR-30b-3p (**Figure 3A**). Then, a dual-luciferase reporter assay showed that miR-30b-3p decreased the luciferase activity of the wildtype (wt) 3′-UTR of TRIM27 compared with the control group, whereas the luciferase activity of the TRIM27 mutant (mut) 3′-UTR group was not significantly different, demonstrating that TRIM27 was the direct target of miR-30b-3p (**Figures 3B**, **C**). To further verify the target relationship between miR-30b-3p and TRIM27, miR-30b-3p mimics or miRNA NC mimics were transfected into the HCC cells, and the RT-qPCR results showed that miR-30b-3p substantially reduced the mRNA level of endogenous TRIM27 compared with the control group (**Figure 3D**). Additionally, downregulating miR-30b-3p expression by anti-miR-30b-3p significantly increased the mRNA level of endogenous TRIM27 (**Figure 3D**). Further, we performed RT-qPCR to assess the mRNA levels of TRIM27 in 50 paired HCC tissues and their adjacent noncancerous tissues. Results showed that the mRNA levels of TRIM27 were significantly higher in these 50 HCC tissues compared with that in their adjacent noncancerous tissues (**Figure 3E**). Lastly, we found that the expression level of miR-30b-3p in these 50 paired HCC tissues and their adjacent noncancerous tissues was negatively correlated with the mRNA levels of TRIM27 (**Figure 3F**). Taken together, these results suggest that miR-30b-3p could downregulate TRIM27 by targeting its 3′-UTR.

### miR-30b-3p Acted as a Tumor Suppressor by Downregulating TRIM27

**Figure 4:**
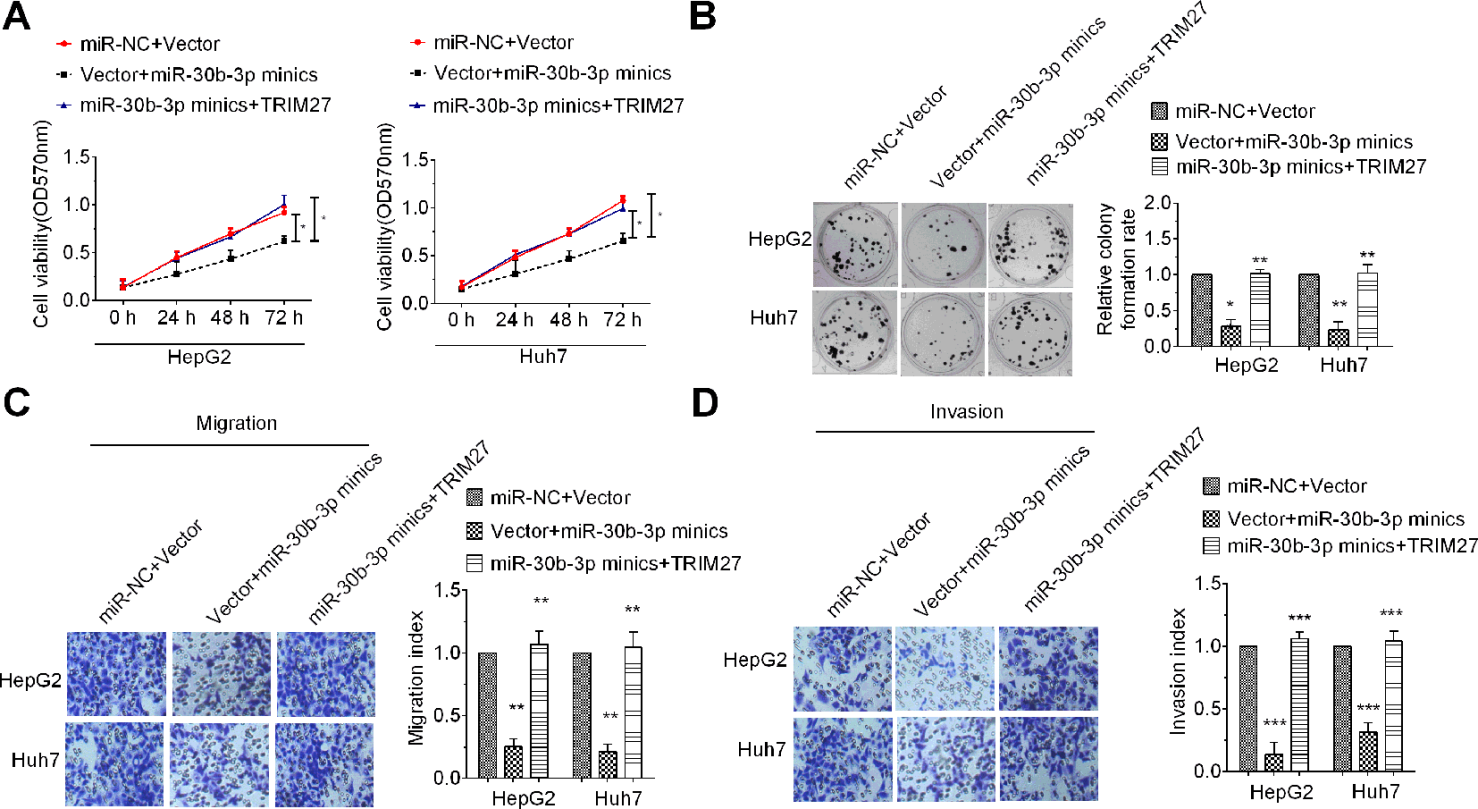
Upregulation of TRIM27 partly rescued the tumour-inhibitory effect mediated by miR-30b-3p. Cell viability **(A)**, colony formation **(B)**, Transwell migration **(C)** and Transwell invasion **(D)** were partly rescued after cotransfection with miR-30b-3p mimics in HCC cells. All the experiments were repeated three times. ^*^P < 0.05; ^**^P < 0.01; ^***^P < 0.001; ^****^P < 0.0001.

To determine whether miR-30b-3p could affect HCC cell proliferation, migration, and invasion through inhibition of TRIM27 expression, we performed rescue experiments. Results of the MTT assay, colony formation assay, and Transwell migration and invasion assay revealed that HCC cells co-transfected with miR-30b-3p mimics plus a TRIM27 overexpression vector showed higher cell viability, a higher colony formation rate and greater Transwell migration and invasion ability, compared with the group co-transfected with miR-30b-3p mimics plus empty vector, indicating that the suppressive role of miR-30b-3p overexpression on the malignant phenotypes of HCC cells was partly counteracted by TRIM27 overexpression (**Figures 4A–D**). These results demonstrated that overexpression of miR-30b-3p inhibited the malignant phenotypes of HCC cells through downregulation of TRIM27.

### TRIM27 Promoted Cell Proliferation, Migration, and Invasion

**Figure 5:**
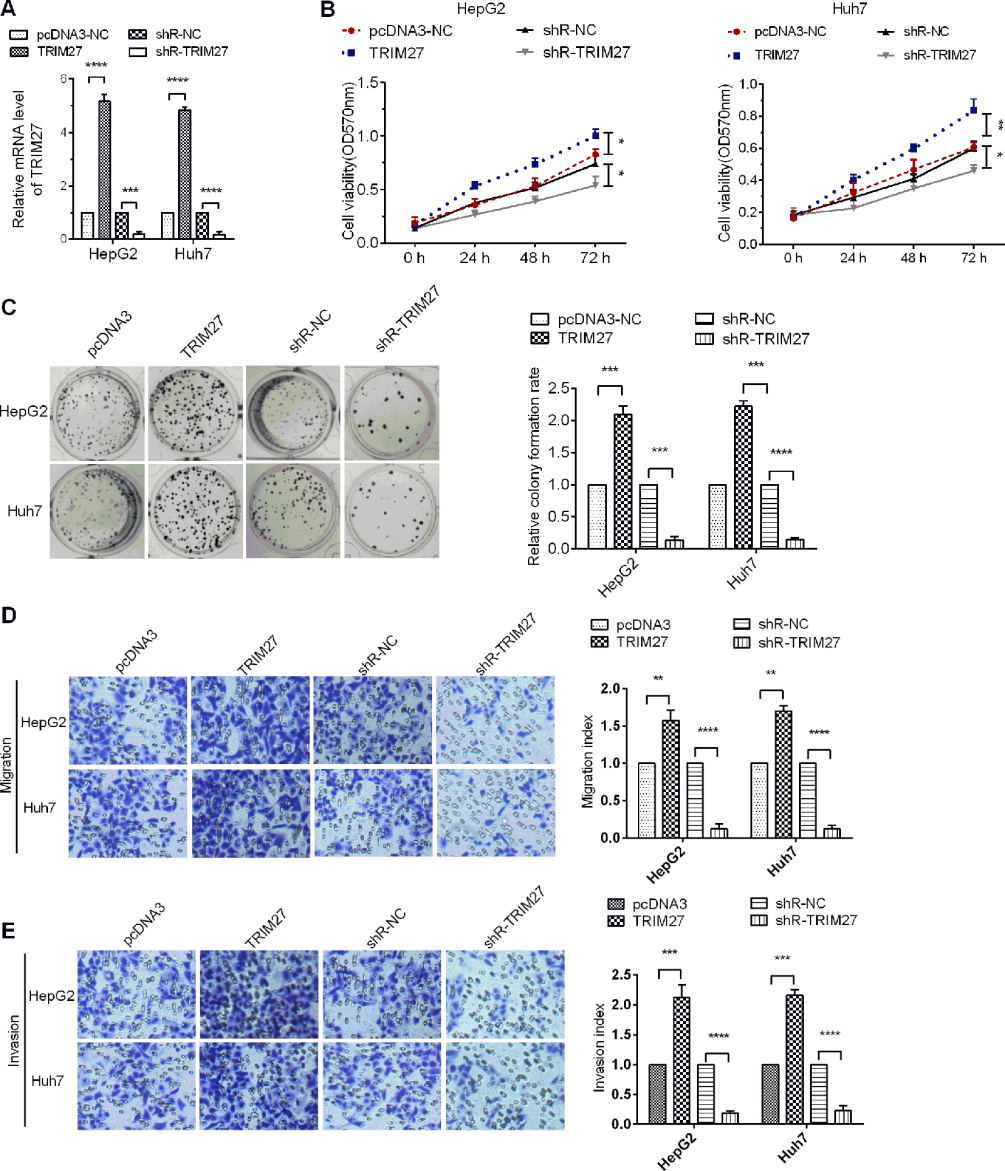
TRIM27 promoted HCC cell proliferation and metastasis. **(A)** RT-qPCR was used to detect the mRNA levels of TRIM27. **(B)** The MTT assay was used to detect the cell viability of HCC cells affected by TRIM27 overexpression or silencing. **(C)** Colony formation in HCC cells was assayed following TRIM27 overexpression or silencing. **(D**, **E)** Representative photographs for cell migration and invasion of HCC cells affected by TRIM27 overexpression or silencing. Data are expressed as mean ± SD, ^*^p < 0.05; ^**^p < 0.01; ^***^p < 0.001; ^****^p < 0.0001.

Previous studies reported that TRIM27 functioned in an oncogenic role in colorectal cancer (Zhang et al., 2018) and ovarian cancer (Ma et al., 2016). However, the role of TRIM27 in HCC remains unclear. To determine the role TRIM27 in HCC cells, TRIM27-overexpressing or -silencing plasmids were transfected into Huh7 and HepG2. The expression of TRIM27 was first detected in HCC cells to verify the efficacy of RIM27-overexpressing or -silencing plasmids by RT-qPCR assays (**Figure 5A**). Then, MTT assay results revealed that TRIM27 overexpression increased cell viability in HCC cells, whereas downregulation of the expression of TRIM27 reduced cell viability (**Figure 5B**). Further, overexpression of TRIM27 markedly enhanced the proliferative effect of HCC cells when compared with the control group, whereas the proliferative effect of HCC cells was significantly inhibited when TRIM27 was silenced (**Figure 5C**). Further, to investigate the role of TRIM27 in HCC cell migration and invasion, the Transwell migration and invasion assay revealed that, compared with the empty vector group, TRIM27 overexpression promoted the malignant phenotypes of HCC cells remarkably, whereas TRIM27 knockdown decreased the migratory and invasive activities of HCC cells (**Figures 5D**, **E**). These results implied that TRIM27 might play an oncogene role in HCC cells.

### miR-30b-3p Inhibited Cell Proliferation, Migration, and Invasion by Targeting the TRIM27/PI3K/Akt-Signalling Pathway

**Figure 6:**
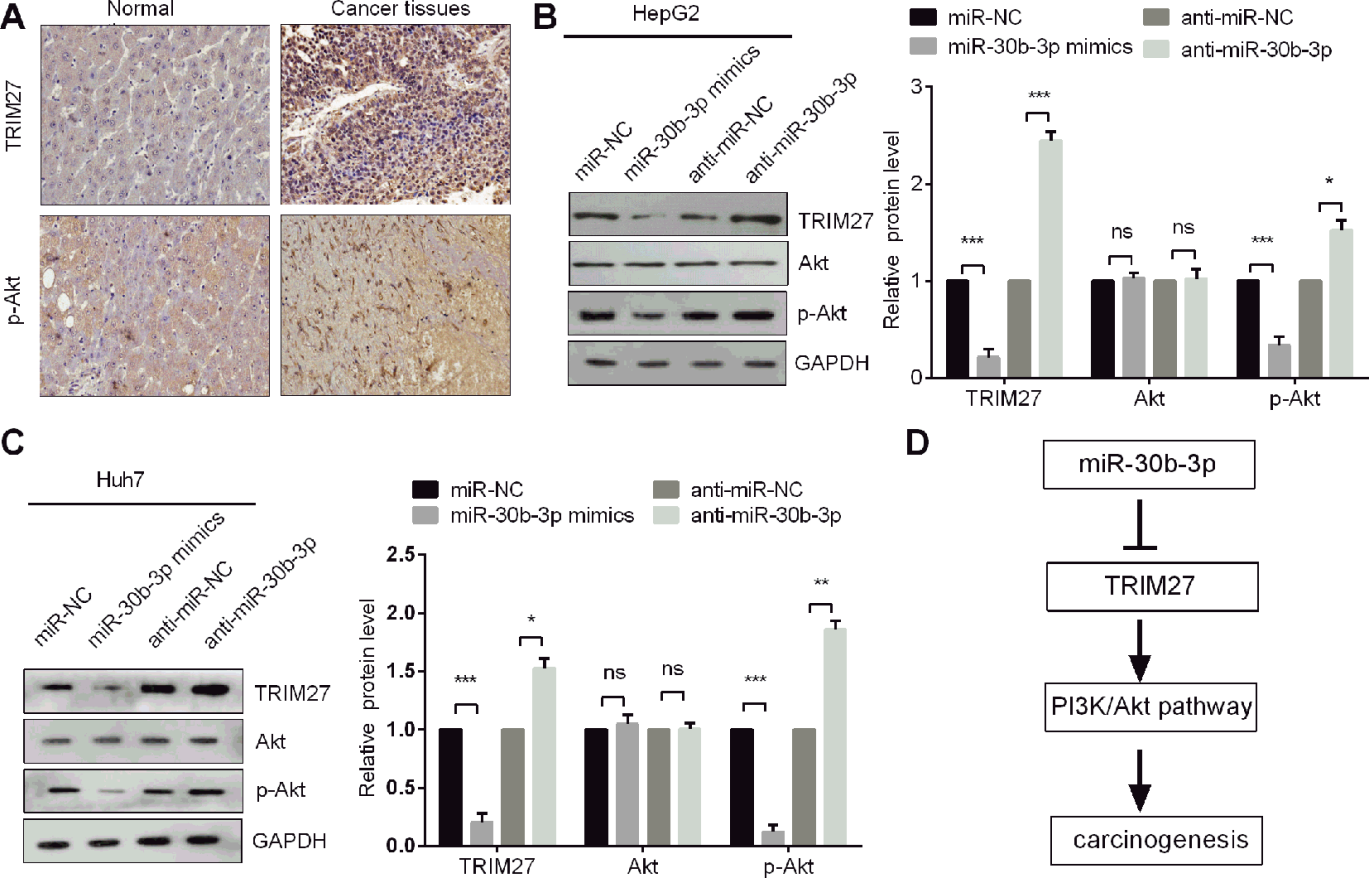
miR-30b-3p inhibited the PI3K/Akt pathway in HCC cells. **(A)** IHC staining of TRIM27 and p-Akt in HCC tissues and their matched non-carcinoma tissues. **(B**,**C)** Western blotting was used to detect the protein levels of TRIM27, Akt, and p-Akt in HCC cells after transfection with indicated transfections. **(D)** The proposed mechanism indicated that miR-30b-3p suppressed the PI3K/Akt pathway by downregulating TRIM27 in HCC cells. All the experiments were repeated three times. ^*^, p < 0.05; ^**^, p < 0.01; ^***^, p < 0.001; ns, not significant.

It was reported that TRIM27 promoted EMT through activation of p-Akt in colorectal cancer (Zhang et al., 2018). The PI3K/Akt signalling pathway is one of the well-characterised pathways in cancer progression (Frezzato et al., 2019). To determine whether miR-30b-3p could affect PI3K/Akt signalling, we first observed that the protein levels of TRIM27 and p-Akt were both highly expressed in HCC tissues compared with non-carcinoma tissues (**Figure 6A**), indicating that PI3K/Akt signalling might be activated when TRIM27 is overexpressed HCC tissues. Further, we demonstrated that miR-30b-3p overexpression reduced the protein level of p-Akt, whereas silencing miR-30b-3p increased the p-Akt levels, which indicated miR-30b-3p could inactivate PI3K/Akt signalling (**Figures 6B, C**). Taken together, our results demonstrated that miR-30b-3p could inhibit the carcinogenesis of HCC through repression of TRIM27/PI3K/Akt signalling (**Figure 6D**).

## Discussion

An increasing number of studies have unveiled the crucial role of miRNAs in many human diseases, from cardiovascular diseases to cancers (Alvarez-Garcia and Miska, 2005; Ikeda et al., 2007; Hebert and De Strooper, 2009; De Santa et al., 2013; Wojciechowska et al., 2017). Evidence has shown that miR-30b-3p acts as a tumour suppressor in PCa, but the biological function of miR-30b-3p in HCC is still ambiguous. In this study, we first confirmed that miR-30b-3p was downregulated in HCC tumor tissues and cell lines. Moreover, analysis by means of the Kaplan–Meier plotter online tool showed that HCC patients with highly expressed miR-30b-3p exhibited longer survival than those with low expression of miR-30b-3p. Previously, Lupold and Kung et al. illustrated that miR-30b-5p can act as a tumour suppressor in the progression of PCa (Kao et al., 2014; Kumar et al., 2016). Although Zeng et al. reported that miR-30b-3p played an oncogene role in glioma cells (Jian et al., 2019), our results were consistent with the former points of view supported by Lupold and Kung et al. (Kao et al., 2014; Kumar et al., 2016).

Herein, we first demonstrated that miR-30b-3p repressed HCC cell proliferation, migration, and invasion in HCC cells. Further, we also uncovered the possible mechanism of miR-30b-3p in HCC cells. As we know, miRNAs usually performed their functions by binding to the 3′-UTR of target genes. To verify the putative targets of miR-30b-3p, we performed the online bioinformatics software TargetScan Human 7.2 and dual-luciferase reporter assay and demonstrated that miR-30b-3p could directly target TRIM27, which has been further verified to be upregulated in 50 HCC tissues compared with that in their adjacent noncancerous tissues and negatively correlated with the levels of miR-30b-3p, suggesting that miR-30b-3p could inhibit HCC cell proliferation, migration, and invasion by downregulating TRIM27.

TRIM27 was first characterised as an oncogene involved in activation of the RET proto-oncogene by DNA rearrangement (Takahashi et al., 1985). Previous studies have been demonstrated that TRIM27 was upregulated in several cancers, such as lung, ovarian, germ cell, endometrial, and breast cancers (Tezel et al., 2002; Tezel et al., 2009; Tsukamoto et al., 2009; Horio et al., 2012; Iwakoshi et al., 2012). It has been shown that TRIM27 plays a multifunctional role in regulating cell proliferation, apoptosis, and the innate immune response (Hatakeyama, 2011). Recent study has shown that TRIM27-activated STAT3 to promote colitis and colitis-associated carcinogenesis (Zhang et al., 2018). In addition, Fu et al. illustrated that TRIM27 promotes EMT through activating p-Akt in colorectal cancer (Zhang et al., 2018). Our study demonstrated that TRIM27 was upregulated and might play an oncogene role in HCC cells. Moreover, we demonstrated that miR-30b-3p could inhibit the carcinogenesis of HCC through repression of TRIM27/PI3K/Akt signalling.

## Conclusion

Our study demonstrated that miR-30b-3p was downregulated in HCC tissues and miR-30b-3p repressed HCC cell proliferation, migration, and invasion in HCC cells by repressing TRIM27/PI3K/Akt signaling. Our findings provide a potential diagnostic and therapeutic target for HCC treatment.

## Data Availability Statement

The raw data supporting the conclusions of this article will be made available by the authors, without undue reservation, to any qualified researcher.

## Ethics Statement

The studies involving human participants were reviewed and approved by the Institutional Ethics Committee of Zhuji People’s Hospital of Zhejiang Province. The patients/participants provided their written informed consent to participate in this study.

## Author Contributions

DG designed the research, performed the experiments, and wrote the manuscript. ZZ and HH were responsible for data collection and statistical analysis.

## Conflict of Interest

The authors declare that the research was conducted in the absence of any commercial or financial relationships that could be construed as a potential conflict of interest.
